# The alternation of neural oscillations during dual task standing in older adults with low back pain

**DOI:** 10.1186/s11556-025-00378-7

**Published:** 2025-07-15

**Authors:** Le Ge, Yao Zu, Zhicheng Li, Xin Li, Huanjie Huang, Yan Li, Xiaoyu Gao, Xi Chen, Qiuhua Yu, Chuhuai Wang

**Affiliations:** Department of Rehabilitation Medicine, The First Affiliated Hospital, Sun Yat-sen University, Guangzhou, China

**Keywords:** Low back pain, Older adults, Electroencephalography, Brain oscillations, Dual-task

## Abstract

**Background:**

Previous studies showed that the difficulty levels of posture and cognitive tasks and pain could interactively modulate the brain oscillations. Older adults with low back pain (LBP) have poorer postural control than healthy older adults under dual-task conditions. However, the underlying mechanism remains unclear. Hence, this study aimed to investigate alterations in brain activation during dual tasks in older people with LBP.

**Methods:**

This cross-sectional study involved older participants with LBP (*n* = 21) and healthy older adults (*n* = 18) without a history of LBP. Electroencephalogram data and balance performance data were recorded simultaneously during dual and single tasks that required the participants to maintain stability in posture tasks with or without a concurrent cognitive task. The posture tasks had two levels of difficulty: a two-leg stance and one-leg stance. Cognitive tasks involved three levels of difficulty: no-cognition tasks, counting tasks, and arithmetic tasks. Brain activities were assessed using the power spectral density (PSD) of alpha-, beta-, and theta-band power rhythms within three regions of interest including the frontal, central, and parietal regions of the brain.

**Results:**

A repeated-measures analysis of variance (2 postural tasks × 3 cognitive tasks × 2 groups) was used to test balance performance, cognitive performance and brain activities under different task conditions between the two groups. Compare to controls, LBP participants showed poorer performance in postural tasks (reflected by larger COP parameters) and cognitive tasks (reflected by lower accuracy rates) regardless of task difficulty level (*p* < 0.05). LBP participants showed larger COP parameters in the dual task with high and low cognitive difficulties than those in single task (*p* < 0.05), which was not observed in control group. The theta band power of control group was higher during one-leg stance than during two-leg stance in frontocentral regions (*p* < 0.05), which was not observed in LBP group. The LBP group showed greater beta-band power in the frontal regions than the control group in all conditions(*p* < 0.05). Correlations between COP parameters and theta band power in frontal regions were significant in dual task or one-leg stance(*p* < 0.05).

**Conclusions:**

In older people with LBP, the brain oscillations as assessed on the PSD of beta and theta power rhythms is changed under the dual-task condition compared with control group. Cognitive and postural difficulty levels could modulate theta band power in frontal region, which subsequently affected the balance performance in older people with LBP.

**Supplementary Information:**

The online version contains supplementary material available at 10.1186/s11556-025-00378-7.

## Background

Low back pain (LBP) is the most common type of pain experienced in older adults [1]. LBP is a significant threat to their motor functions and daily activities, which subsequently leads to falls and a decline in quality of life [2]. Older adults with LBP have higher rates of falls than healthy individuals due to the poor postural control [3, 4]. Postural tasks (e.g., standing) are commonly accompanied by cognitive tasks (e.g., talking, reading, or calculating) in daily life [5]. In our previous study, we found that older people with LBP had poor postural control during concurrent postural and cognitive tasks, regardless of the difficulty of the tasks, thereby leading to a higher risk of falling [6]. However, healthy older adults only showed poor postural control in the postural task with higher difficulty cognitive task, but not in that with lower difficulty cognitive task [6]. Xiao et al. [7] demonstrated that individuals with high levels of pain exhibited poor behavior performance and increased difficulty maintaining balance when performing cognitive tasks concurrently with motor tasks. Therefore, it is substantial to explore the underlying neural mechanism during dual tasks in older adults with LBP, which could help design more precise treatment program.

Various human neuroimaging studies have used magnetic resonance imaging (MRI), functional near-infrared spectroscopy, and electroencephalography (EEG) to investigate brain activation and cortical involvement in the upright posture during concurrent postural and cognitive tasks [8–10]. A widespread decrease in absolute alpha band power reflects the global processes of increased attention and alertness. However, activities in the sensorimotor and parietal cortical regions appear to be associated with movement and sensory-related information processing, especially in the condition of standing postural control [11]. Alpha band power (8–13 Hz) in both the sensorimotor and parietal cortices decreases with increased difficulty during a single task (postural task only) and dual tasks (i.e., postural task and serial subtraction task) in healthy adults [11, 12]. In addition, increased alpha power has been noted in frontal, sensory, temporal, and parietal areas in chronic pain patients in resting state compared to healthy controls [13]. Theta band power was found to contribute to pain modulation. For instance, increased theta activity has been reported in participants with chronic pain, both in resting state as well as task conditions [14]. The increase in theta frequency band power within the frontal and central regions may also reflect high-level cognitive process including encoding and retrieval [15, 16]. For instance, compared to single task, the power of the theta band (5–7 Hz) in the frontal, central, and parietal regions increased under dual-task conditions (i.e., an upright stance and a 2-back task) [17]. In addition, the theta band power in frontal and sensorimotor cortex region were strongly related to balance performance and fall risk [18]. The beta band power in the frontal and central regions, however, was related with higher self-perception pain intensity when conducting the functional tasks [19]. The patients with chronic pelvic pain showed increased beta band power, which was related to the pain intensity [20]. All these findings suggested that the difficulty levels of posture and cognitive tasks and pain could interactively modulate the brain oscillations. However, the previous studies more explored the behavioral performance in postural control performance in single or dual tasks and not take the difficulty level of task into consideration. The neural mechanism underlying concurrent postural and cognitive tasks in older adults with LBP is yet to be elucidated.

This study aimed to investigate alterations in brain activation during dual tasks in older adults with LBP, using EEG spectral power analysis of the alpha, beta, and theta bands. We hypothesized that poorer balance performance/cognitive performance would be observed in the dual task than single task in LBP group compared to the healthy control group. With comparison to healthy older adults, those with LBP would show decreased alpha power and increased theta power which are linked to cognitive process and postural control; the power in the beta band linked to pain perception would increase due to the long-term pain.

## Methods

### Study design and participants

Older participants with LBP (LBP group) and healthy participants as control group were recruited from local community and different older activity centers by posting the advertisement. The sample calculation was performed by using G*Power Software (v3.1.9.7) to determine the sample size by testing within-between interaction. We estimated the sample size according to our previous research [6]. The balance data of sway area was selected as primary outcome variable. With a power analysis set power of 0.80, а of 0.05 and a large effect size (Cohen’s f = 0.185), 17 participants were required for each group. The study protocol was approved by the ethics committee of our hospital (number#2019469), in accordance with the Declaration of Helsinki and informed consent was obtained from individuals prior to participation. The inclusion criteria for LBP group are as follows: (1) age (60–85 years); (2) nonspecific LBP for at least 3 months in the previous year; (3) pain score > 3/10 on the Visual Analog Scale (VAS); and (4) Mini-Mental State Examination (MMSE) score > 24(out of 30)and Montreal Cognitive Assessment (MoCA) score ≥ 26 (out of 30) As a control group, healthy adults were included who fulfilled the following criteria: (1) age (60–85 years); (2) MMSE score > 24(out of 30) and Montreal Cognitive Assessment (MoCA) score ≥ 26 (out of 30); and (3) no history of LBP in the past 12 months. Participants in both the groups were excluded if they (1) had a history of spinal or lower-extremity surgery, endocrine or neuromuscular disease, spinal tumors, rheumatological diseases of the spine, arthritis or orthopedic disease, orthostatic hypotension, vision or vestibular system disease, or any other physical injury; (2) used psychoactive or antihypertensive drugs (antidepressants, antipsychotics, sedatives/hypnotics, antiepileptics, antiparkinsonian drugs); (3) had severe postural abnormalities; (4) Edinburgh Handedness Inventory score less than 40 [21].

### Experimental procedure

**Fig. 1:**
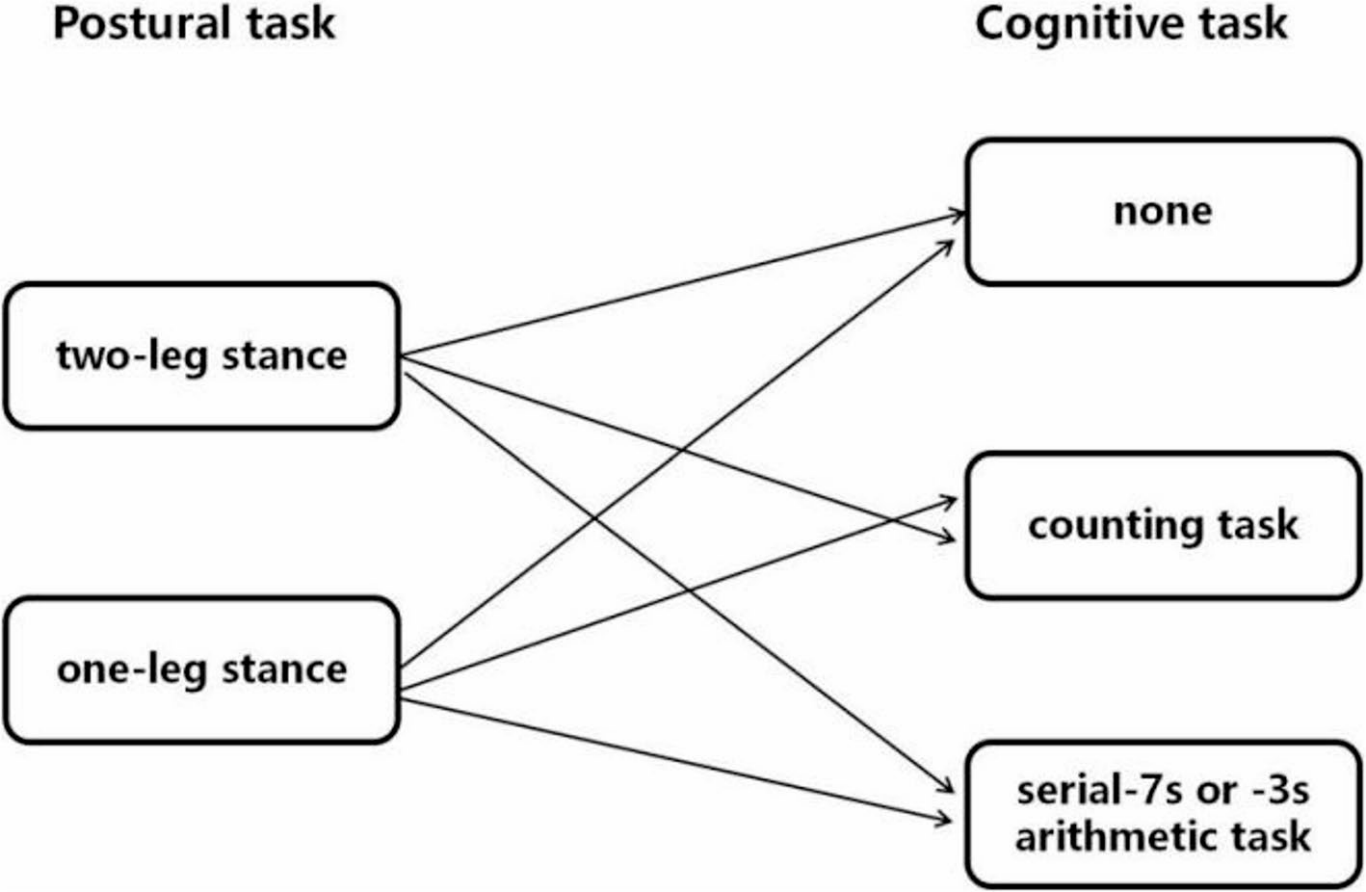
Combinations of postural tasks and cognitive tasks

At the beginning of the experiment, the participants completed an information sheet that included demographic data and number of falls in the past 12 months. Demographic data included weight, height, body mass index (BMI), and education level. Fall was defined as unintentionally coming to rest on the ground, floor, or other level with or without an injury [22]. The LBP group initially completed the 10-cm VAS and the Oswestry Disability Index (ODI) questionnaires and then completed a dual task (i.e., concurrent postural and cognitive tasks) and a single task in a dimly lit and soundproof EEG chamber. During the dual and single tasks, each participant was asked to stand on a Nintendo Wii Balance Board with barefoot and look ahead at the center of a computer screen, 80 cm in front of their eyes. At the beginning, the participants were required to practice several times until they fully understood the task. In the single-task condition, the participants were required to perform the postural task alone, whereas in the dual-task condition, they were required to perform the postural task along with a cognitive task (counting and calculation) (Fig. 1). All the conditions in single and dual tasks were assigned randomly to participants and the order was determined by the random function in the Microsoft Office Excel 2007. During the dual and single tasks, the center of pressure (COP) displacements and Electroencephalogram (EEG) data were simultaneously recorded. The total experiment time was approximately 1 h.

### Postural task

**Fig. 2:**
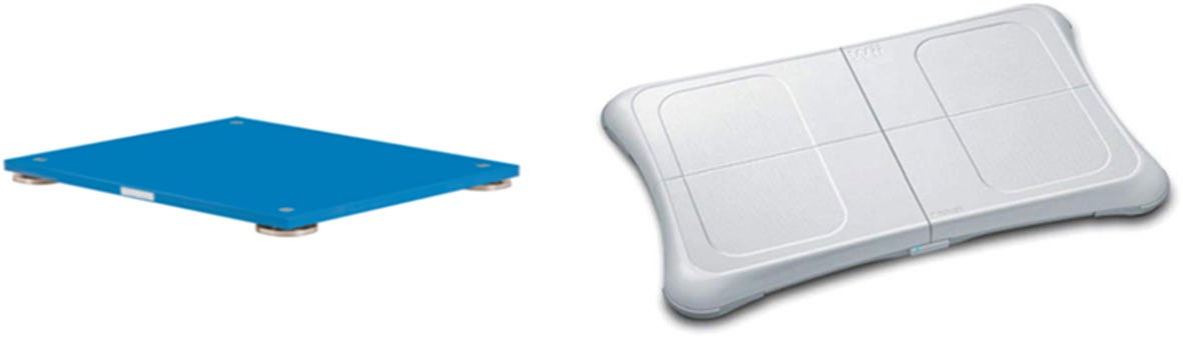
Wii Balance Board

The postural task could be divided into two conditions of different difficulty while standing on the Nintendo Wii Balance Board (WBB; Nintendo, Kyoto, Japan) connected by bluetooth to a laptop computer was used to record COP displacements; (i) two-leg stance; (ii) one-leg stance. We used the Nintendo Wii Balance Board (WBB) to assess balance function. The WBB is equipped with four force sensors placed at the four corners of the board that are used to evaluate the vertical ground reaction forces (Fig. 2). The following formulas are used to detect body sway in the left-right (COPx) and anterior-posterior (COPy) directions:

$$\begin{aligned}COPx=&\frac{Lx}{2}*(\left(TR+BR\right)-\left(TL+BL\right))\\&/(TL+TR+BL+BR)\end{aligned}$$

$$\begin{aligned}COPy=&\frac{\text{L}\text{y}}{2}\text{*}(\left(TL+TR\right)-\left(BL+BR\right))\\&/(TL+TR+BL+BR)\end{aligned}$$

where TL, TR, BL, BR represent the four force sensors in the four corners of the force plate (Top Left, Top Right, Bottom Left, Bottom Right), Lx, Ly represent the length of the WBB in the x and y dimensions which are 45 cm and 27.5 cm, respectively [23].These sensor values are transmitted wirelessly to the Wii console with a sampling frequency of 100 Hz.Studies have shown that the sway length and sway area obtained from WBB are validated outcome parameters to reflect the postural stability [24].All COP trajectories were calculated expressed in mm, sway area (mm2) and sway length(mm/s) were used as the outcome variables in this study.

### Cognitive task

**Fig. 3:**
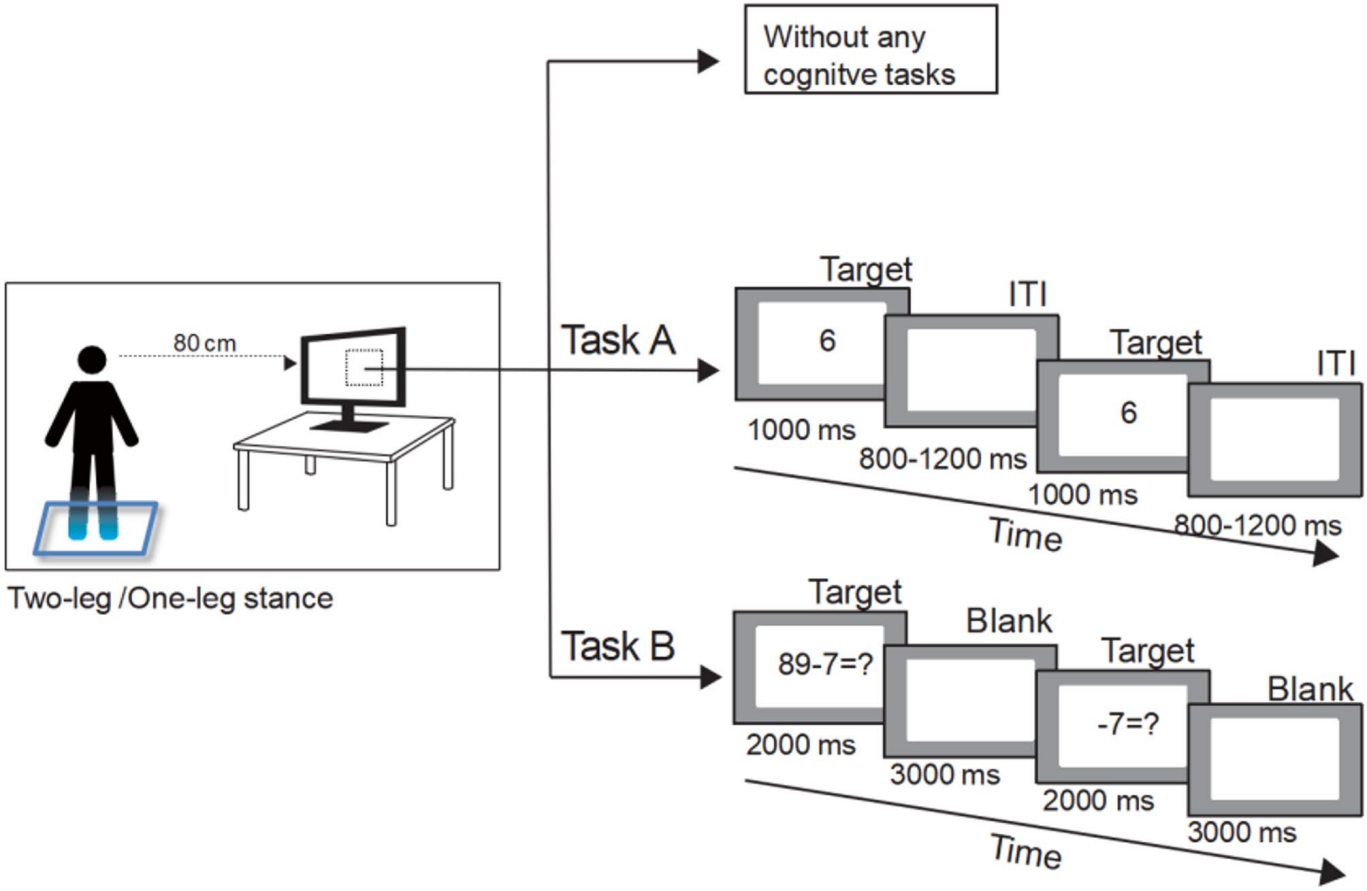
Illustration of the task design. All tasks are performed on a dimly-lit and soundproof EEG chamber. The experiment consists of three tasks, which are (1) two-leg stance or one-leg stance without cognitive task; (2) postural task combined with counting task (Task A); and (3) postural task combined with serial-7s or serial-3s arithmetic task (Task B)

The cognitive task consisted of two subtasks of two difficulty levels. The subtask with the lowest difficulty was the counting task (Task A). In the counting task, numbers 1 to 9 were randomly shown to the participants. The participant was then required to count the number of times the target number was displayed on the computer monitor and provide an answer as soon as possible after the last number in each block was displayed (Fig. 3, Task A), thus the counting task (Task A) required the involvement of sustained attention and working memory. In each trial, one number from 1 to 9 was randomly presented for 1000 ms, followed by a blank inter-stimulus interval of 800 to 1200 ms. There were three blocks with 20 trials in each block. Each block lasted approximately 40s. The other subtask with high difficulty was a “serial-7s or serial-3s arithmetic task” (Task B), which made reference to that in our previous study [6]. In one block of the serial-7s or-3s arithmetic task, the participant was required to start with any number from 89 to 100 and then subtract 7 or 3 several times. There were three blocks with 6–7 trials in each block. In each block, the target stimulus of the first trial was an equation subtracting 7 or 3, with minutes randomly selected from 89 to 100. The target stimulus of the first trial lasted for 2000 ms, followed by a blank interval for 3000 ms. The second target stimulus of the succeeding trials after the first trial showed “−7=?” for 2000 ms, also followed by a blank interval of 3000 ms (Fig. 3, TaskB). During the subtraction, the participants were required to keep the subtraction result of the previous trial in mind to complete the subtraction in the next trial, which required a greater cognitive load than the counting task. The participants were asked to provide the last answer as fast as possible at the end of each block. The accuracy rates in the sitting (as the baseline of cognitive performance) and standing positions were used in subsequent data analysis. All the stimuli in both cognitive tasks were displayed using E-prime 2.0.

### EEG preprocessing

Electrocortical activity was recorded using 64 passive saline based sponge electrodes (RNET; Brain Products, Germany). The actiCHamp Plus system with passive electrodes included an amplifier, batteries, and R-Net (electrode caps based on saltwater sponges and passive Ag/AgCl electrodes). EEG data were recorded using a BrainVision Recorder and analyzed using the BrainVision Analyzer software (Brain Products, Germany). The R-Net of the 64-channel cap follows the international 10–20 positioning system. The electrode distribution is shown in Fig. 3. Before starting the EEG recording, impedances were set to be lower than 20 kΩ for all electrodes. The sampling rate was 1000 Hz. Channels Tp9 and Tp10 were selected as the reference electrodes. After re-referencing to the average of Tp9 and Tp10 and filtering (0.01–30 Hz), independent component analysis was conducted to remove the ocular artifact. Then the EEG data were segmented every 2 s (epochs). After that, we removed the epochs with an amplitude exceeding ± 80 µv and the trials with incorrect answer. Finally, a fast Fourier transformation was applied to the EEG data after artifact rejection to obtain the power spectral density at three frequencies including alpha (8–13 Hz), beta (14–30 Hz), and theta (4–7 Hz).

### Statistical analysis

An independent t-test was used to determine the differences in age, height, weight, MMSS score, MoCA score, and BMI between the LBP and control groups. The chi-square test was used to determine between-group differences in falls in the past 12 months. There was significant between-group difference in BMI and weight (Table 1). As this variable was associated with cognition and balance [25, 26], BMI and weight was used as a covariate in the statistical analysis. By adjusting for the covariates of BMI, the power spectral density of three predefined frequency bands of three brain regions and cognitive performance in single or dual tasks were assessed using a mixed model analysis of covariance (ANCOVA) with the covariates of BMI. Three brain regions were frontal (F3-F1-Fz-F2-F4), central (C3-C1-Cz-C2-C4), and parietal (P3-P1-Pz-P2-P4) [27]. The between-subjects factor was group (LBP vs. control). The within-subject factors were postural difficulty (two-leg stance versus one-leg stance) and cognitive difficulty (none vs. low difficulty versus high difficulty). Post-hoc pairwise comparisons with Bonferroni adjustments were applied for significant main or interaction effects. The Greenhouse–Geisser correction was used if Mauchly’s test of sphericity was violated. Given that the theta band power was strongly related to balance control and fall risk [18], the theta band power in the present study was selected to explore the associations between brain activity and COP parameters. The Shapiro–Wilk test was used to check the distribution of continuous variables. The results of Shapio-Wilk test showed that the data of theta band power and COP parameters were normally distributed. Then Pearson correlation analysis was used to explore the relationship between theta band power and COP parameters, with the level of significance set at *p* < 0.05. All statistical analyses were performed using SPSS v25.0 (IBM Corp., Armonk, NY, USA).

## Results

### Participant characteristics

21 older adults with LBP were recruited in the LBP group and 18 participants for the control group. The clinicodemographic participant characteristics are presented in Table 1. All the participants were right-handed. There were no significant between-group differences with respect to age, sex, weight, height, or education (all *p* > 0.05). Meanwhile, BMI and number of falls in the past 12 months were higher in the LBP group than in the control group (all *p* < 0.05) (Table 1).

**Table 1:** Demographic characteristics of the two groups

Item	LBP (*n* = 21)	control (*n* = 18)	t/χ^2^	*p*
Age(years)	65.14 (4.48)	64.50 (3.07)	0.513	0.611
Sex	Female(17)	Female(15)	0.037	0.847
Height(m)	1.62 (7.66)	1.60 (6.87)	0.534	0.597
Weight(kg)	60.33(7.72)	56.16 (4.73)	1.98	0.05
BMI (kg/m2)	22.89 (2.0)	21.73 (1.75)	1.90	0.06
Pain duration (years)	7.38(6.42)	Not applicable		
VAS(the highest)	8.47(1.03)	Not applicable		
ODI(%)	16.38(10.83)	Not applicable		
The frequency of falls in the past 12 months	0(8)	0(15)	—	0.023
	1(7)	1(3)	—	
	2(5)	2(1)	—	
	3(1)	3(0)	—	
Education(years)	≥ 10(15)	≥ 10(12)	—	0.799
	5–10(3)	5–10(4)	—	
	≤ 5(3)	≤ 5(2)	—	

Noted: VAS and ODI, are shown as median (interquartile range); The frequency of falls in the past12 months are expressed as number of falls (number of person); Education years (number of person); other outcome variables are shown as mean (standard deviations) LBP, low back pain; VAS, visual analog scale; ODI, Oswestry disability index

### Postural performance

Table 2 shows the changes of COP parameters in the task with different combinations of postural difficulty and cognitive difficulty. Table 3 presents a summary of ANCOVA results for all data of postural performance in the single or dual tasks.

Significant between-group difference were found in sway area [F(1,37) = 61.781, *p* < 0.001, ƞ2p = 0.625] and sway length [F(1,37) = 105.354, *p* < 0.001, ƞ2p = 0.740]. The main effects of postural difficulty were significant in sway area [F(1,37) = 1028.273,*p* < 0.001, ƞ^2^*p* = 0.965] and sway length [F(1,37) = 657.486, *p* < 0.001, ƞ2p = 0.947]. The main effects of cognitive difficulty were also significant in sway area [F(2,74) = 41.731, *p* < 0.001, ƞ2p = 0.530] and sway length [F(1.479,68.269) = 27.234, *p* < 0.001, ƞ2p = 0.424].

The group×postural difficulty×cognitive difficulty effect was significant in sway area [F(1.913,70.780) = 6.225, *p* = 0.004, ƞ2p = 0.144], sway length [F(2,74) = 10.312, *p* = 0.003, ƞ2p = 0.218]. Post hoc analysis showed that the LBP group had larger sway length and sway area than the control group in the dual task (*p* < 0.05) but not in the single task, when the condition was two-leg stance. But the LBP group showed larger sway length than the control group in both the single and dual tasks (*p* < 0.05) in one-leg stance. The group × postural difficulty effects were significant in all COP parameters sway area [F(1,37) = 6.765, *p* = 0.013, ƞ^2^*p* = 0.155] and sway length [F(1,37) = 10.778, *p* = 0.002, ƞ^2^*p* = 0.226]. Post hoc analysis showed that the LBP group had larger COP parameters than the control group in two levels of postural difficulties (*p* < 0.05). The group ×cognitive difficulty effects were only significant in sway area [F(1.845,68.296) = 4.02, *p* = 0.025, ƞ^2^*p*=0.098]. Post hoc analysis for the group × cognitive difficulty effects showed that the LBP group had larger sway area than control group in three levels of cognition difficulties (*p* < 0.05). LBP participants showed larger COP parameters in the dual tasks with high and low cognitive difficulties than those in single task (*p* < 0.05), which was not observed in control group. These results suggested that compared to the healthy older people, the older people with LBP had poor postural performance reflected by larger COP parameters regardless of any postural or cognitive difficulties. Compared with the single task, the postural control of LBP group was impaired in the dual task, even though the difficulty level of the cognitive task was low.

**Table 2:** Balance performance in different combinations of postural difficulty and cognitive difficulty for two groups

Condition		Sway area Mean (SD)		Sway length Mean (SD)	
		LBP	Control	LBP	Control
Single task	Two-leg stance	235.71(94.64)	240.77(89.81)	316.42(94.83)	335.00(85.62)
	One-leg stance	1067.0(185.42)	886.16(90.81)	1119.7(209.76)	889.72(65.62)
Dual task	Two-leg stance + taskA	397.80(71.77)	273.94(73.77)	399.28(57.22)	300.38(54.01)
	Two-leg stance + taskB	537.95(103.79)	325.83(54.85)	515.14(69.61)	371.55(76.31)
	one-leg stance + taskA	898.28(73.52)	734.27(94.38)	974.09(78.08)	754.50(109.06)
	one-leg stance + taskB	1236.0(164.99)	1149.7(120.52)	1293.0(169.52)	1085.7(105.88)

Noted: *SD* denotes standard deviation

task A: counting task, task B: serial-7s or serial-3s arithmetic task

## The EEG activity

**Fig. 4:**
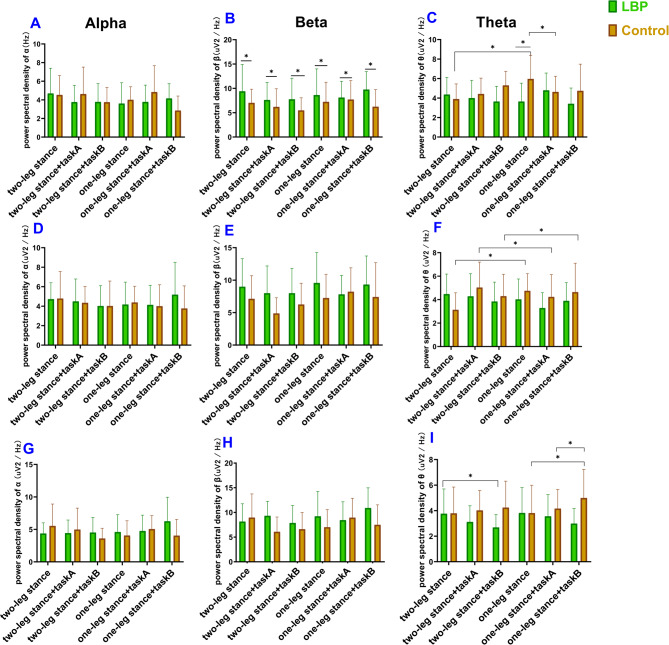
Power spectral density of alpha, beta, and theta in the frontal, central, and parietal regions. **a-c** Power spectral density of alpha, beta, and theta in the frontal region. **d-f** Power spectral density of alpha, beta, and theta in the central region. **g-i** Power spectral density of alpha, beta, and theta in the parietal region. Error bar indicates standard deviation.  Indicates significant difference within-group for post hoc analysis.  Indicates significant difference between-group for post hoc analysis

The mean power spectral density at the three frequencies (i.e., alpha, beta, and theta bands) in different combinations of postural and cognitive tasks in the frontal, central, and parietal regions are shown in Fig. 4. Table 3 presents a summary of the statistical results of power spectral density at the three frequencies (i.e., alpha, beta, and theta) in the frontal region. The theta band had significant effect in the group × postural difficulty× cognitive difficulty interaction (F = 3.761, *p* = 0.033, ƞ^2^*p*=0.181). However, the beta band only showed significant group main effects (F = 4.893, *p* = 0.033, ƞ^2^*p*=0.276). The beta band power in the LBP group was higher than control group regardless the postural and cognitive tasks difficulty (Table 4), indicating beta band could be modulated by pain. Post hoc analysis of the group × postural task × cognitive task interaction of theta band power displayed that LBP group showed lower theta band power than the control group in the one-leg stance of single task (*p* < 0.05) (Fig. 4). For within-group comparisons, the theta-band power in the control group was higher during one-leg stance than during two-leg stance under a single task (*p* = 0.002). However, there were no significant differences between the two cognitive or postural tasks in the LBP group (*p* > 0.05).

**Table 3:** The summary of ANCOVA results for all data of power spectral density of alpha, beta, theta in frontal region

Item	alpha			beta			theta		
	F	*p*	ƞ^2^*p*	F	*p*	ƞ^2^*p*	F	*p*	ƞ^2^*p*
Main effect									
Group	0.031	0.861	0.001	4.893	0.033	0.120	4.535	0.040	0.115
Postural difficulty	2.283	0.140	0.061	0.491	0.488	0.014	1.299	0.262	0.036
Cognitive difficulty	1.534	0.223	0.042	1.594	0.210	0.077	0.887	0.416	0.025
Interaction effect									
Group × postural difficulty	0.299	0.588	0.008	0.637	0.430	0.017	3.876	0.057	0.100
Group × cognitive difficulty	3.046	0.061	0.094	0.176	0.831	0.012	1.859	0.163	0.050
Postural difficulty × cognitive difficulty	0.519	0.600	0.015	0.681	0.508	0.039	0.258	0.773	0.007
Group × postural difficulty× cognitive difficulty	0.976	0.387	0.021	0.427	0.653	0.021	3.761	0.033	0.103

**Table 4:** Power spectral density of beta in the frontal across different combinations of postural difficulty and cognitive difficulty between the two groups

Condition		Power spectral density of beta Mean (SD)	
		LBP	control
Single task	Two-leg stance	9.40(5.25)	7.03(2.80)
	One-leg stance	8.63(5.37)	7.23(4.04)
Dual task	Two-leg stance + task A	7.62(3.60)	6.19(3.73)
	Two-leg stance + task B	7.76(4.33)	5.47(2.59)
	one-leg stance + task A	8.12(3.34)	7.71(3.88)
	one-leg stance + task B	9.76(3.69)	6.23(3.25)

Noted: SD denotes standard deviation; task A: counting task, task B: serial-7s or serial-3s arithmetic task

The statistical results of power spectral density at the three frequencies (i.e., alpha, beta, and theta) in the central region are presented in Table 5. The mixed model analysis of covariance showed that the alpha band power had a significant main effect in the cognitive task (F = 4.193, *p* = 0.023, ƞ^2^*p*=0.193), while the theta band had a significant interaction effect in the group × postural difficulty (F = 4.506, *p* = 0.041, ƞ^2^*p*=0.111). The results of post hoc analysis of the group × postural difficulty interaction of the theta band showed that theta band power was higher in the control group in the one-leg stance than in the two-leg stance, regardless of the cognitive task difficulty (*p* < 0.05), which was not observed in the LBP group (*p* > 0.05) (Fig. 4).

The summary of the statistical results for power spectral density at the three frequencies (including alpha, beta, theta) in the parietal region is presented in Table 6. The results showed that the theta band had significant group main effect (F = 8.879, *p* = 0.005, ƞ^2^*p*=0.198) and significant interaction effect on group × cognitive difficulty (F = 3.452, *p* = 0.043, ƞ^2^*p*=0.165). Post hoc analysis of the group × cognitive difficulty interaction of the theta band power showed that in the control group, the theta band power was higher under the dual-task condition than under the single-task condition (Fig. 4). In contrast, the LBP group showed lower theta band power under the dual-task condition than under the single-task condition (*p* < 0.05) (Fig. 4). All these EEG results suggested that the older adults with LBP showed disassociated performance in alpha, beta and theta bands of brain oscillations during the posture task with and without cognitive task.

**Table 5:** The summary of ANCOVA results for all data of power spectral density of alpha, beta, theta in central region

Item	alpha				beta			theta			
	F	*p*	ƞ^2^*p*	F		*p*	ƞ^2^*p*	F	*p*	ƞ^2^*p*	
Main effect											
Group	0.110	0.742	0.003	3.369		0.075	0.086	1.185	0.284	0.032	
Postural difficulty	2.402	0.130	0.063	0.954		0.335	0.026	0.638	0.430	0.017	
Cognitive difficulty	4.193	0.023	0.193	2.540		0.086	0.115	0.296	0.745	0.012	
Interaction effect											
Group × postural difficulty	0.002	0.962	0.000	2.820		0.102	0.073	4.506	0.041	0.111	
Group × cognitive difficulty	0.615	0.453	0.082	0.469		0.607	0.020	0.819	0.445	0.040	
Postural × cognitive difficulty	1.305	0.277	0.063	0.972		0.383	0.058	0.655	0.523	0.043	
Group × postural × cognitive difficulty	0.099	0.905	0.007	2.362		0.101	0.117	1.621	0.205	0.106	

**Table 6:** The summary of ANCOVA results for all data of power spectral density of alpha, beta, theta in parietal region

Item	alpha			beta			theta		
	F	*p*	ƞ^2^*p*	F	*p*	ƞ^2^*p*	F	*p*	ƞ^2^*p*
Main effect									
Group	0.179	0.675	0.015	2.450	0.162	0.064	8.879	0.005	0.198
Postural difficulty	0.433	0.515	0.002	1.627	0.210	0.043	0.406	0.528	0.011
Cognitive difficulty	0.528	0.592	0.022	0.037	0.964	0.002	0.038	0.963	0.003
Interaction effect									
Group × postural difficulty	0.820	0.371	0.022	1.193	0.282	0.032	0.125	0.726	0.003
Group × cognitive difficulty	2.404	0.098	0.130	0.805	0.451	0.042	3.452	0.043	0.165
Postural × cognitive difficulty	2.740	0.071	0.126	1.529	0.224	0.097	0.426	0.655	0.019
Group × postural × cognitive difficulty	1.435	0.245	0.061	2.437	0.095	0.113	0.374	0.689	0.010

### Cognitive performance

**Fig. 5:**
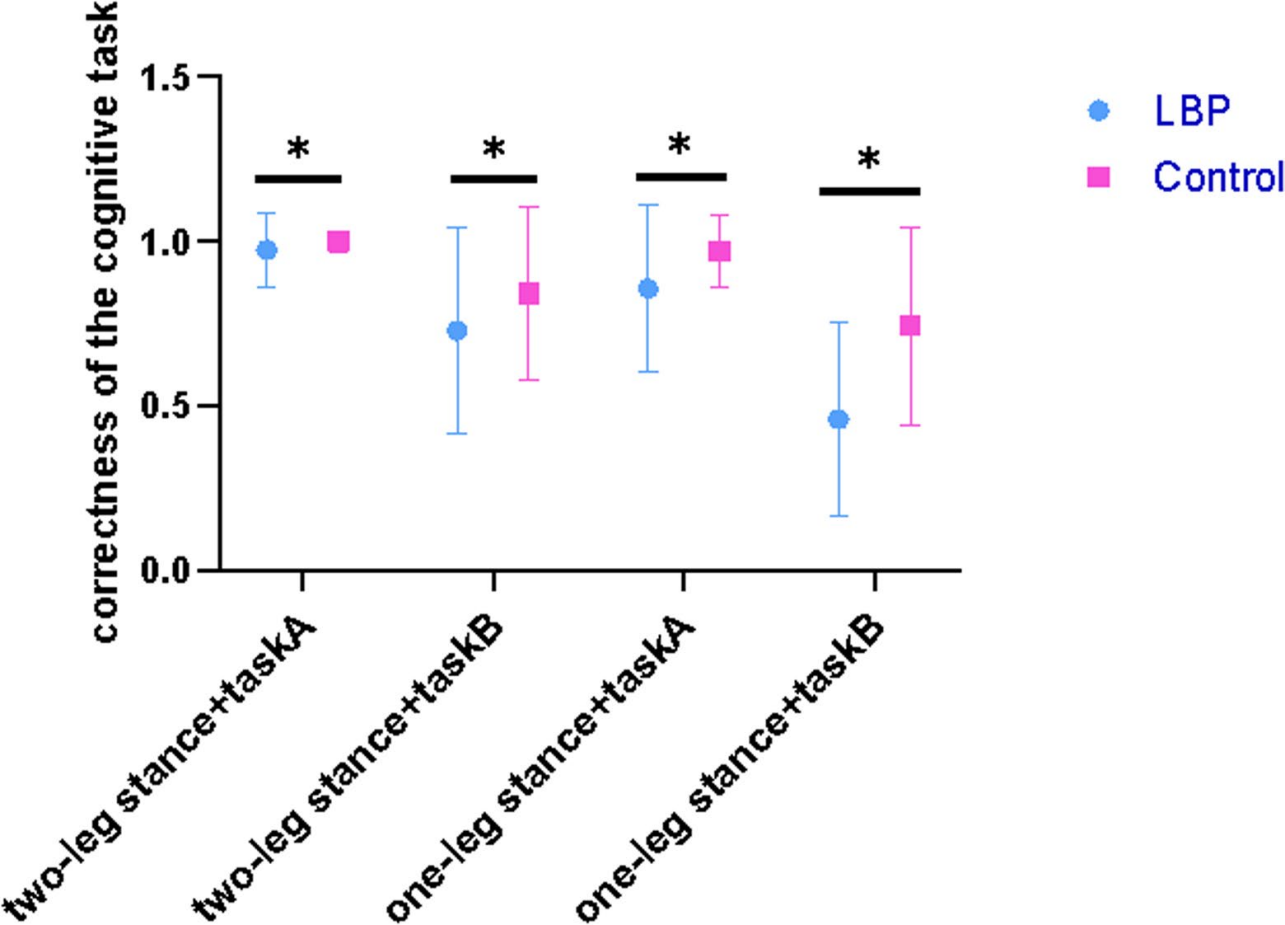
The accuracy rates of the cognitive task in dual task condition. task A: Counting task; Task B: Serial-7s or serial-3s arithmetic task.  means significance difference between two groups

A summary of the statistical results for the cognitive performance of the two groups is shown in Table 7. The results showed that the main effects were significant in group, cognitive difficulty, and postural difficulty. The interaction effects were significant in the group × postural difficulty (F = 4.194, *p* = 0.048, ƞ^2^*p*=0.104). Post hoc analysis of the accuracy rates in the group × postural difficulty showed that the accuracy rates were lower in both groups when the difficulty of the postural task increased (*p* < 0.05). The accuracy rates in the control group were higher than those in the LBP group (*p* < 0.05) regardless of the difficulty level of the postural and cognitive tasks (Fig. 5). The between-group difference of accuracy rates of cognitive task was larger in one-leg stance than two-leg stance.

Associations between the power spectral density (theta in frontal) and COP parameters.

Pearson correlation analysis the relationship between theta band power in the frontal region and COP parameters, which were shown in Table 7. Significant associations between sway length (*R* = −0.336) and theta band power in frontal at a single task were shown only in the single task (*p* < 0.05).The correlations between sway length/sway area and the theta band power in the frontal region at dual task (postural task combined with serial-7s or serial-3s arithmetic task) were significant during two-leg stance (*R* = −0.550) and one-leg stance (*R* = −0.378) conditions (*p* < 0.05). The other associations between the theta band power in the frontal region and the COP parameters were not significant (*p* > 0.05).

**Table 7:** Correlation analysis between theta band power in frontal region and COP parameters

		**two-leg stance**		**one-leg stance**		**two-leg stance+task ** **A**		**one-leg stance+task ** **A**		**two-leg stance+task ** **B**		**one-leg stance+taskB**	
		**sway area**	**Sway length**	**sway area**	**sway length**	**sway area**	**sway length**	**sway area **	**sway length**	**sway area**	**sway length**	**sway area**	**sway length**
power spectral density (theta in frontal region)	r	0.146	0.102	−0.298	−0.336	0.021	0.087	−0.211	0.180	−0.110	−0.550	−0.378	−0.303
	*p*	0.404	0.559	0.082	0.048	0.897	0.599	0.225	0.300	0.530	0.001	0.025	0.077

Noted: task A: counting task; task B: serial-7s or serial-3s arithmetic task

## Discussion

This study is the first to examine neural mechanism underlying concurrent postural and cognitive tasks in older adults with LBP. The main findings of this study showed that LBP participants showed poorer performance in postural control and cognitive tasks regardless of task difficulty level. LBP participants showed larger COP parameters in the dual tasks with high and low cognitive difficulties than those in single task, which was not observed in control group. As for brain activation, compared with healthy controls, older adults with LBP showed alterations in the power spectral density of the alpha, theta, and beta frequency bands during postural tasks with and without concurrent performance of the cognitive task, which are the new findings in the present study.

In accordance with our hypothesis, compared to the healthy control group, older adults with LBP showed poorer balance performance and cognitive performance in dual task than single task condition. Compared with the single task, the balance performance of participants with LBP became worse in the dual task, even though the difficulty level of the cognitive task was low (task A). As for control group, however, was decreased only if the difficulty level of the cognitive task was high. This is consistent with our previous study [6]. The potential reason was complexed, first, fear of falling has an impact on postural control (larger COP parameters) through psychological and behavioral mechanisms, such as increased anxiety, cautiousness behavior and changes in attentional focus [28]. Second, some previous studies found attention deficits in the older adults with LBP [29, 30]. LBP would weaken the balance performance through altered neuromuscular activities and motor control of trunk muscles in the postural task combined with cognitive task [7], especially when the difficulty of cognitive task is high. The reason wat that it was not enough attentional resources to complete postural task or cognitive task in dual tasks for the participants with LBP [6, 31]. That is to say, LBP would interplay with neuromuscular activities and attentional demands. All of these factors were associated with poor balance performance or cognitive performance in older adults with LBP [32]. However, in the present study fear and falling and emotion were not assessed, which should be considered in the future study.

The beta band power in the frontal region was higher in the LBP group than in the control group regardless of the cognitive and postural tasks. These findings were supported by the previous studies. For instance, Teixeira et al. [33] found that beta power in the frontocentral regions was positively associated with self-reported pain intensity in patients with LBP. Increased beta power appears to be related to a compensatory mechanism of greater neuronal injury and represents a subtype of potentially higher central sensitization in response to chronic pain [34]. A previous study reported a negative relationship between beta oscillations and cortical activation during motor tasks [35]. In the present study, the LBP group showed higher beta power than the control group, regardless of the postural or cognitive tasks the participants engaged in. This was most likely to be related to motor dysfunction and long-term pain in these patients.

However, in our study, the theta band power was lower in the LBP group than in the control group in the one-leg stance condition, which was inconsistent with our hypothesis and also contrary to previous study [14] The potential reason was that in previous study, increased theta band power was found in evoked chronic clinical pain conditions or at resting-state EEG conditions in the LBP group. While in our study the EEG was recorded during the postural task or the postural task combined with the cognitive task condition. In the previous study, theta band power, especially in frontal region, is positively related to the cognitive workload during cognitive tasks [36]. Theta power was increased when individuals were engaged in challenging balance conditions, including perturbations, visual occlusion, and additional cognitive load [36, 37].Studies using high-density EEG have shown that the activation of the theta power in the frontal and central regions increases when the difficulty of the posture task increases [38]. These evidences could support our findings in control group. In present study, we found that in the control group, the theta band power in the frontal region was higher in the one-leg stance than in the two-leg stance. And in the central region indicated higher activation of theta power in the one-leg stance than in the two-leg stance regardless of the cognitive task difficulty. The potential reason for the decreased theta band power in the LBP group than control group in the one-leg stance task might be that the participants were unable to recruit additional neural resources to cope with real-world challenges. One-leg stance was more challenging than two-leg stance, which required more cognitive resources to cope with the challenges [39]. However, our results that the LBP group did not show any differences in the theta band in frontal and central regions with increasing difficulty in the postural task or cognitive task. The potential reason was that pain-related cognitive fatigue or motivational factors could contribute to reduced theta activation under dual task condition [40], and long-term pain also causes theta hypoactivation under dual-task condition [41].

In the parietal region, the theta band power showed that in the control group, the theta band power was higher under dual-task condition than under the single-task condition. In contrast, the LBP group showed lower theta band power under the dual-task condition than under the single-task condition. Higher theta band power under dual-task condition in control group was consistent with those reported in the previous study [16]. The power of the theta band in old adults increased in the frontal, central, and parietal regions under dual-task condition due to higher cognitive resources required in the dual task [14]. The previous study reported that activation of theta power in the parietal region is associated with error processing in cognitive and motor tasks [12, 17]. But when the difficulty of the postural or dual task increased, the LBP group had decreased neural-related activation in parietal region. These findings are consistent with the extensive shift in brain organization and morphology observed in neuroimaging studies explored by transcranial magnetic stimulation, MRI, and voxel-based morphometry in patients with LBP [42–44].Previous studies on postural control during maintain standing balance in response to rotations of the support surface tasks have demonstrated that the area of cortical activation is change in subjects with LBP, the enhanced cortical of postural challenge is part of an adaptive compensation. Thus, in our study the alternation in cortical activation in these patients may indicate altered pain-processing mechanisms compared to healthy individuals.

Previous research has reported that frontal activity is important for balance control, which further supports the increasing number of studies emphasizing the involvement of frontal theta oscillations in balance control [18]. Our study also found the between-group differences of theta band power in frontal region. Therefore, the present study explored the associations between theta band power in the frontal region and COP parameters. It has been reported that higher demands on balance control are associated with an increase in frontal midline theta power. In our study significant associations in the single task were found only in the one-leg stance and in the dual-task with higher cognitive difficulty. Task such as one-leg stance and dual-task paradigm being more challenging in daily life which need more involvement of frontal theta oscillations to maintain balance control. In this study, compare with control group, LBP people showed poorer balance control (large COP parameters) in the one-leg stance and dual-task conditions, while their theta band power was lower in the one-leg stance and dual-task conditions. When the postural task was high or combined with cognitive task, the cognitive and postural tasks compete with each other owing to limited cognitive resources. Unlike the control group, the LBP group may not be able to allocate more cognitive resources to compensate for cognitive disruption in the postural control system [32]. Competition for shared cognitive resources during dual tasks might increase the cognitive-motor interference for postural control and/or cognitive task performance, which subsequently lead to falls or poor balance in older adults with LBP.

LBP is an important risk factor of repeated falls in older adults [45], where LBP significantly contributes to the number of years spent living with a disability, which in turn leads to poor balance and falls [2]. However, the underlying brain mechanisms remain unclear. To our best knowledge, this study was the first to examine brain oscillations in older adults with LBP while performing a balance task with an increased task difficulty level, with and without cognitive tasks. The brain activity alterations in the brain regions in this study might represent the long-term pain that leads to altered neural processing for postural control in older adults with LBP, so that LBP patients seem unable to recruit additional neural resources to cope with real-world challenges [32]. This might be the reason for poor balance control or falls during daily activities in patients with LBP.

Although the present study revealed novel findings about the neural correlates of cognitive versus postural interference, our study had some limitations. A major limitation of the present study is we only used frequency spectrum analysis to examine the neural mechanisms in older adults with LBP in the dual-task condition. Future studies should employ event related potential and electromyography to explore the underlying neuromuscular mechanism at specific time point. Secondly, the majority participants in each group were female. The results in our study might be not generalized to the male older adults. Future study should recruit the participants with a more balanced sex distribution.

## Conclusions

Older adults with LBP showed impaired postural control during cognitive and postural dual task, which might be related to the altered brain oscillations, associated with poor balance control or falls during daily activities. Our findings shed light on the interaction of pain and cognitive capacity on postural control and its underlying mechanism. Motor-cognitive dual task training might be the potential rehabilitation intervention for enhancing the postural control of older adults with LBP.

## Supplementary Information

Supplementary Material 1.

## Data Availability

No datasets were generated or analysed during the current study.

## Abbreviations

ANCOVA: analysis of covariance
BMI: body mass index
EEG: electroencephalography
LBP: low back pain
MMSE: Mini-Mental State Examination
MoCA: Montreal Cognitive Assessment
MRI: magnetic resonance imaging
ODI: Oswestry Disability Index
PSD: power spectral density
VAS: Visual Analog Scale
